# Supplementary material for: A randomized, double-blind pilot study of analgesic and anti-inflammatory effects of naproxen sodium and acetaminophen following dental implant placement surgery
Source: Front Pharmacol. 2023 May 17;14:1199580. doi: 10.3389/fphar.2023.1199580 (PMC10229806; doi:10.3389/fphar.2023.1199580)
Supplement: Supplementary file 1 [file Table1.DOCX]

Supplementary Material

A Randomized, Double-Blind Pilot Study of Analgesic and Anti-Inflammatory Effects of Naproxen Sodium and Acetaminophen Following Dental Implant Placement Surgery

Katherine N. Theken*, Mengxiang Chen, D. Lucas Wall, Truongan Pham, Stacey A. Secreto, Thomas H. Yoo, Allison N. Rascon, Yu-Cheng Chang, Jonathan M. Korostoff, Claire H. Mitchell, Elliot V. Hersh

*** Correspondence:** Katherine N. Theken, PharmD, PhD: ktheken@pennmedicine.upenn.edu

**Supplementary Table 1. Dosing schedule of study medication employed to maintain the blind**

|  | Naproxen Sodium Group | Acetaminophen Group |
| --- | --- | --- |
| **Day One:**  Immediately post-surgery  6 hours post-surgery  12 hours post-surgery | 220 mg x 2  Placebo x 2  220 mg x 1 plus placebo x 1 | 500 mg x 2  500 mg x 2  500 mg x 2 |
| **Day Two**  On Awakening  6 hours post dose one  8 hours post dose one  12 hours post dose one  16 hours post dose one | 220 mg x 1 plus placebo x 1  Placebo x 2  220 mg x 1 plus placebo x 1  Placebo x 2  220 mg x 1 plus placebo x 1 | 500 mg x 2  500 mg x 2  Placebo x 2  500 mg x 2  Placebo x 2 |
| **Day Three**  On Awakening  6 hours post dose one  8 hours post dose one  12 hours post dose one  16 hours post dose one | 220 mg x 1 plus placebo x 1  Placebo x 2  220 mg x 1 plus placebo x 1  Placebo x 2  220 mg x 1 plus placebo x 1 | 500 mg x 2  500 mg x 2  Placebo x 2  500 mg x 2  Placebo x 2 |

**Supplementary Table 2.** Inpatient effectiveness rating and global evaluation

|  | Naproxen sodium (n=15) | Acetaminophen (n=15) |
| --- | --- | --- |
| Effectiveness Rating |  |  |
| Mostly or Completely Effective | 15 (100%) | 10 (66.7%) |
| Somewhat Effective | 0 (0%) | 2 (13.3%) |
| Somewhat Ineffective | 0 (0%) | 2 (13.3%) |
| Completely or Mostly Ineffective | 0 (0%) | 1 (6.7%) |
| Global Evaluation ^†^ |  |  |
| Poor | 0 (0%) | 0 (0%) |
| Fair | 0 (0%) | 1 (6.7%) |
| Good | 1 (7.7%) | 3 (20.0%) |
| Very Good | 4 (30.8%) | 5 (33.3%) |
| Excellent | 8 (61.5%) | 6 (40%) |

† Global evaluation was not collected for 2 naproxen-treated patients who did not attend the 72-hour follow-up visit

**Supplementary Table 3.** Peak inflammatory mediator concentrations by clinical and demographic characteristics

|  | Gender | | Body Mass Index | | Number of Implants | |
| --- | --- | --- | --- | --- | --- | --- |
|  | Men (n=12) | Women (n=18) | <25 kg/m^2^ (n=12) | >25 kg/m^2^ (n=18) | 1 implant (n=24) | 2 implants (n=6) |
| Plasma IL-6 (pg/ml), T=6 hr | 12.3±9.3 | 15.5±14.4 | 14.6±13.2 | 13.9±12.4 | **11.5±9.7** | **25.0±17.5 p=0.10** |
| GCF IL-1β (pg/ml), T=24 hr | 893±889 | 722±928 | **277±295** | **1204±1011 p<0.05** | 730±882 | 1148±981 |
| GCF IL-8 (pg/ml), T=24 hr | 4952±3636 | 4698±4624 | **2382±2839** | **6822±3707**  **p<0.05** | 4480±3786 | 6968±5031 |

**Supplementary Figure 1.** CONSORT flow diagram


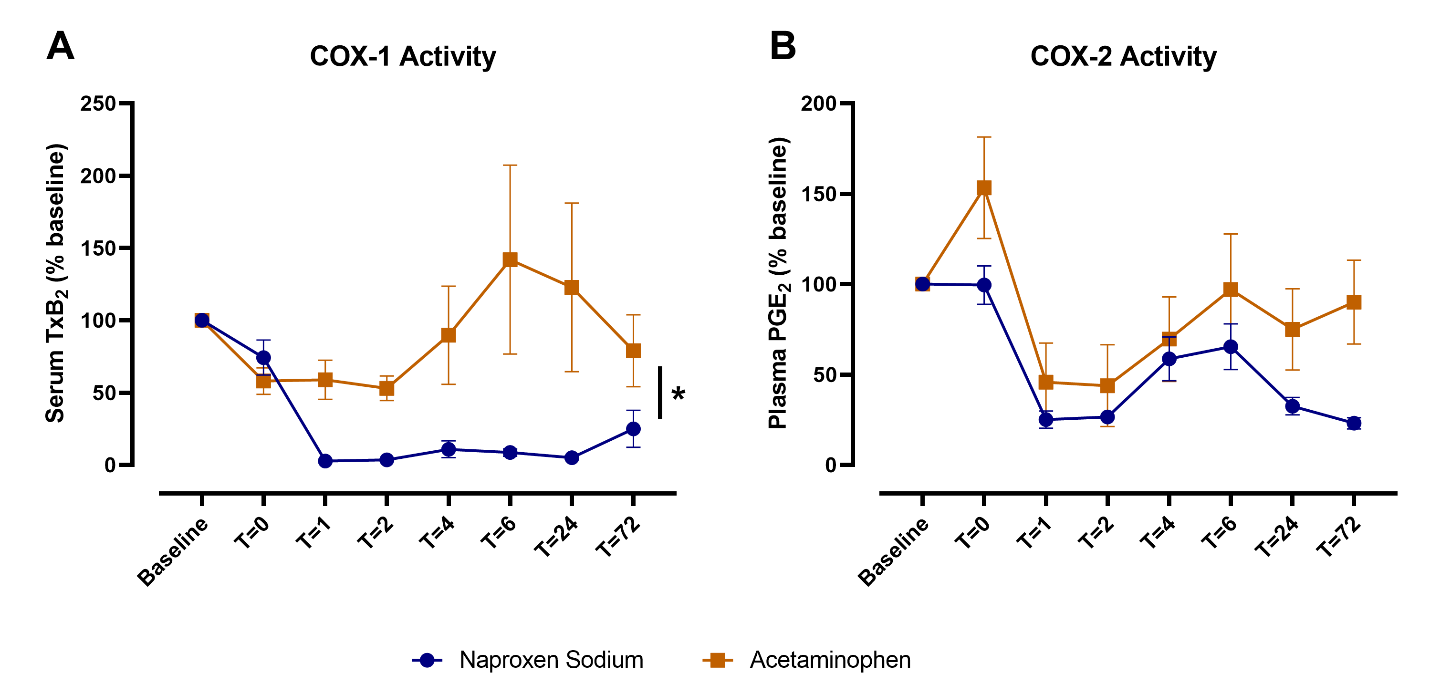


**Supplementary** **Figure 2.** Comparison of (A) COX-1 and (B) COX-2 activity over time between patients treated with naproxen sodium (blue) and acetaminophen (orange). Data are expressed as a percent change from baseline and shown as mean ± SEM (*p<0.05 for time x treatment interaction).
